# Significance of logistic regression scoring model based on natural killer cell-mediated cytotoxic pathway in the diagnosis of colon cancer

**DOI:** 10.3389/fimmu.2023.1117908

**Published:** 2023-01-20

**Authors:** Zhen Ye, Huanhuan Zhang, Jianwei Liang, Shuying Yi, Xianquan Zhan

**Affiliations:** ^1^ Medical Science and Technology Innovation Center, The Second Affiliated Hospital of Shandong First Medical University, Jinan, Shandong, China; ^2^ Department of General Surgery, Tai ‘an Central Hospital, Taian, Shandong, China

**Keywords:** ULBP2, logistic regression, immunohistochemistry, immune, IC50

## Abstract

**Background:**

The poor clinical accuracy to predict the survival of colon cancer patients is associated with a high incidence rate and a poor 3-year survival rate. This study aimed to identify the poor prognostic biomarkers of colon cancer from natural killer cell-mediated cytotoxic pathway (NKCP), and establish a logistical regression scoring model to predict its prognosis.

**Methods:**

Based on the expressions and methylations of NKCP-related genes (NRGs) and the clinical information, dimensionality reduction screening was performed to establish a logistic regression scoring model to predict survival and prognosis. Risk score, clinical stage, and ULBP2 were used to establish a logistic regression scoring model to classify the 3-year survival period and compare with each other. Comparison of survival, tumor mutation burden (TMB), estimation of immune invasion, and prediction of chemotherapeutic drug IC50 were performed between low- and high-risk score groups.

**Results:**

This study found that ULBP2 was significantly overexpressed in colon cancer tissues and colon cancer cell lines. The logistic regression scoring model was established to include six statistically significant features: S = 1.70 × stage – 9.32 × cg06543087 + 6.19 × cg25848557 + 1.29 × IFNA1 + 0.048 × age + 4.37 × cg21370856 − 8.93, which was used to calculate risk score of each sample. The risk scores, clinical stage, and ULBP2 were classified into three-year survival, the 3-year prediction accuracy based on 10-fold cross-validation was 80.17%, 67.24, and 59.48%, respectively. The survival time of low-risk score group was better than that of the high-risk score group. Moreover, compared to high-risk score group, low-risk score group had lower TMB [2.20/MB (log10) vs. 2.34/MB (log10)], higher infiltration score of M0 macrophages (0.17 vs. 0.14), and lower mean IC50 value of oxaliplatin (3.65 vs 3.78) (p < 0.05).

**Conclusions:**

The significantly upregulated ULBP2 was a poor prognostic biomarker of colon cancer. The risk score based on the six-feature logistic regression model can effectively predict the 3-year survival time. High-risk score group demonstrated a poorer prognosis, higher TMB, lower M0 macrophage infiltration score, and higher IC50 value of oxaliplatin. The six-feature logistic scoring model has certain clinical significance in colon cancer.

## Introduction

Colon cancer is a common malignant tumor, which affects 1.2 million people worldwide and causes ~0.6 million deaths each year (1). Data from the North American Society**’**s Central Cancer Registry revealed that the incidence rate of colon cancer was 27.6 per 100,000 individuals between 2012 and 2016. The incidence rate of distal colon cancer increases annually by 1.5%–1.8% from birth to 65 years of age (p<0.05) (2). Thus, colon cancer is a major socioeconomic burden for human society. The accurate prediction of patient prognosis enables accurate clinical diagnosis and treatment.

Cytotoxicity is the ability to kill other cells, which is an important effector of immune system to fight against viral infections and cancer. Cytotoxic T cells and natural killer (NK) cells are the major mediators of cytotoxic activities (3). NK cells are lymphocytes involved in the innate immune response, which can exert cytotoxic effects on tumor cells (4). Targeting Siglec-7 and -9 are the potential novel therapeutic approach to enhance NK cell responses against cancer (5). NK cells are involved in immune monitoring and tumor development regulation *via* inducing apoptosis in malignant cells. Among the major mechanisms of NK cell-mediated cytotoxicity, the death receptor pathway and the release of particles with perforin/granzyme are the major mechanisms involved in eliminating tumor cells (6). The activation of NK cells using (As + Ce6) @MSNS-PEG, an immune activator, effectively inhibited the proliferation of colon cancer cells *in vitro* and *in vivo* (7).

Recent achievement in science and technology has significantly contributed to precision medicine. Precision medicine involves the accurate assessment of disease risk, diagnosis, prognosis, and treatment response by patient stratification with individual clinical information and biomarkers (8). Thus, precision oncology involves the delivery of optimal customized treatment to the patient at the effective dose and specific time point (9). Many types of nanoparticles that are used for drug delivery in colon cancer facilitate the precise targeting of selective drugs to the tumor site, improve efficiency, and mitigate cytotoxicity (10). Molecular targeted drugs can prolong the survival of metastatic colon cancer patients. Next-generation sequencing enables clinicians to easily identify biomarkers of colon cancer (11).

Colon cancer is a complex disease, its management and control cannot depend on the old “one disease one drug” strategy, and must transit to a new era of predictive, preventive, and personalized medicine (PPPM) (12). However, the accurate judgment of clinical prognosis and survival of colon cancer patients is currently at a nascent stage. Currently, the prognosis and survival of patients with colon cancer are roughly estimated. For example, clinicians provide a general judgment on the prognosis of colon cancer based on its clinical stage.

Several survival and prognosis models have been reported in colon cancer (13–17). However, the prediction accuracy of these models is not high. For example, a prediction model was established by screening a large number of genes in combination with clinical data, whose area under the curve (AUC) of 3-year survival prediction was 0.785 (13). A study established a prognostic model with five ferroptosis-related genes in colon cancer, its AUC of 3-year survival prediction was 0.686 (14). A study used the random forest method and least absolute shrinkage to establish an immune risk score model, its AUC of 3-year survival prediction was 0.691 in validation cohort (15). For another multigene model in colon cancer, its AUC of 3-year survival prediction was 0.774 (16). In addition, a nomogram was established with risk score and TNM classification, which achieved an AUC of 0.872 for 3-year survival prediction, but lacking of independent sample validation (17).

This study used gene expression and methylation sites in NK cell-mediated cytotoxic pathway (NKCP) in combination with clinical information to establish a logistic regression model after dimensionality reduction to obtain a risk score. Subsequently, the logistic regression model was validated with the risk score to predict the 3-year survival of colon cancer patients. The accuracy of the final logistic regression model was tested with 10-fold cross-validation. Survival, enrichment, mutation, and chemotherapeutic drug sensitivity analyses were compared between low- and high-risk score groups. This study aimed to provide a strategy for prediction of prognosis and survival of colon cancer patients.

## Materials and methods

### Transcriptomic and clinical data of colon cancers

The genes derived from NKCP were obtained from the Kyoto Encyclopedia of Genes and Genomes (KEGG) database. Transcriptomic, methylation, mutation, and clinical data of colon cancer were downloaded from The Cancer Genome Atlas (TCGA) database. The transcriptomic data were RNA seq_count data. DNA methylation data were obtained from Illumina Human Methylation 450. Methylation sites with deletions greater than 5% were deleted, while those with deletions less than 5% missing values were filled with “impute” R-package (https://bioconductor.org/packages/release/bioc/html/impute.html). The type of mutation data was selected as masked somatic mutation. To extract patient ID, gender, age, ethnicity, tumor stage, survival time, and final survival status, Python 3.7 was used to import the pandas package (https://pandas.pydata.org/), and the JSON clinical file was downloaded.

### Identification of differentially expressed NKCP-related genes (DENRGs) of colon cancers

DENRG analysis was performed with the “edger” software of the R-package (https://bioconductor.org/packages/release/bioc/html/edgeR.html). The PlotMD () function was used to plot differential distribution of genes of colon cancers. The criteria to screen DENRGs were log fold-change (FC) >1 or <−1 and p<0.05. The heatmap of DENRG expression levels was constructed by “ComplexHeatmap” (https://bioconductor.org/packages/release/bioc/html/ComplexHeatmap.html) of R-package with the Heatmap () function. Differential methylation sites were also analyzed with the “edger” package (threshold>4 times or < 0.25 times, and p < 0.05) (**Supplementary Table 10**).

### Predictive biomarkers of poor prognosis

DENRGs were used for survival analysis to determine the effects of each DENRG on overall survival and prognosis of colon cancers. According to the sequencing value of genes, the patients were roughly divided into 3 subgroups, namely, low-expression group, medium-expression group, and high-expression group. Kaplan-Meier analysis was performed to examine survival contribution of each DENRG. Univariate analysis was performed with the “SurvMiner” package (https://cran.r-project.org/web/packages/survminer/index.html) using the function “surv_fit”. The survival analysis curve was completed with the function “ggsurvplot.”

### Cell culture

Colon cancer cell lines (LoVo and RKO cells) and healthy colon cell line (NCM460 cells) were used to validate the selected high-risk gene (ULBP2) at the mRNA level. All cell lines were cultured in Dulbecco’s modified Eagle’s medium supplemented with 10% fetal bovine serum in a 5% carbon dioxide incubator at 37°C.

### Quantitative PCR analysis of ULBP2

Quantitative PCR (qPCR) was used to measure the mRNA levels of ULBP2 in colon cancer cells vs. control cells. Briefly, total RNAs were extracted from ~10^6^ cultured cells with a total RNA extraction kit (Beyotime Biotechnology Reagent Company). The concentration and purity of the extracted RNAs were determined, with A260/A280 value ranges from 1.9 to 2.0, which show a high-quality RNAs. Subsequently reverse transcription was performed in a 20-µL reaction mixture with 2 µg template RNAs. qPCR was performed in a 10-µL reaction mixture that comprised multiplex RNA master mix (Roche), forward and reverse primers, and complementary DNA (cDNA) template on a Roche LightCycler96 real-time PCR instrument. The expression of target RNA was normalized with reference gene *GAPDH*. The relative expression of the analyzed gene was calculated with 2^−ΔΔ^Ct method (18).

### Immunohistochemical analysis of ULBP2

Fresh tissue specimens (n=9) from colon cancer surgery, Department of General Surgery, Tai ‘an Central Hospital, China, were fixed for 24 h with a universal tissue fixative solution (Wuhan Servicebio Technology Co., LTD, Chian). The primary antibody anti-ULBP2 antibody (Wuhan Servicebio Technology Co., LTD, China) was diluted (1:100) and incubated with the prepared tissues. The stained tissue sections were examined with Olympus microscope (400x), and were scored with criteria: (i) staining depth was ranged from 0 to 4 based on the depth of yellow color; and (ii) staining percentage was calculated with 0 point for <20%, 1 point for 20%–40%, 2 points for 40%–60%, 3 points for 60%–80%, and 4 points for >80%. The values from (i) and (ii) were summed to yield qualitative results: ≤2 points mean negative (-); 3–4 points mean weak positive (+); 5–6 points mean positive (++); and 7–8 points mean strong positive (+++). Moreover, the Human Protein Atlas website (www.proteinatlas.org) was used to determine immunohistochemical staining level of ULBP2 protein in healthy colon tissue.

### Establishment of logistic regression model for risk score

Colon cancer patients were divided into two groups based on the final survival time: groups L (good prognosis; survival time >=3 years), and S (poor prognosis; survival time < 3 years). Logistic regression model was established for the risk score, and this model involved three aspects of features (n=27 features): (i) patient clinical data, including age and tumor stages I (assigning number 1), II (assigning number 2), III (assigning number 3), and IV (assigning number 4); (ii) DENRGs were analyzed with the “Caret” package to identify the top 20 genes with the strongest ability to distinguish between groups L and S for subsequent gene screening; and (iii) differential methylation sites of the NKCP-related genes were analyzed to identify the first five loci that could differentiate group L from group S for subsequent screening. Finally, a logistic regression model was established with glm function of R language based on these 27 features. Each feature with p<0.1 was included in the final logistic regression model. After dimensionality reduction, six features were incorporated into the model, and each sample was scored. Then, the logistic regression model was established again for the score of each sample to classify the samples. The accuracy of classification was compared with the control logistic regression models established with different features. For example, the control 1 model established by clinical staging; Control 2 model was established by ULBP2, and control 3 model was established by clinical stage and patient age. The accuracy of this logistic regression model was verified with 10-fold cross-validation in the Weka (version 3.8.5).

### Determination of high- and low-risk score groups

The median value of risk scores of all samples was used to divide colon cancer samples into high- and low-risk score groups.

### Survival analysis

Prognosis status of high- and low-risk score groups was analyzed with Kaplan-Meier survival analysis. The Cox regression analysis was used to screen risk factors that affect survival. Multivariate survival analysis was performed with “SurvMiner” and “Survival” (https://cran.r-project.org/web/packages/survival/index.html) packages.

### GO and KEGG enrichment analyses of DENRGs

DENRGs between high- and low-risk score groups were subjected to gene ontology (GO) and KEGG enrichment analyses using “enrichplot” (https://bioconductor.org/packages/release/bioc/html/enrichplot.html) software package of R language, including “clusterProfiler” (https://bioconductor.org/packages/release/bioc/html/clusterProfiler.html ), “org.Hs.eg.db”(https://bioconductor.org/packages/release/data/annotation/html/org.Hs.eg.db.html), “GOSemSim”(https://bioconductor.org/packages/release/bioc/html/GOSemSim.html), “DOSE”(https://bioconductor.org/packages/release/bioc/html/DOSE.html), and “ggplot2”(https://cran.r-project.org/web/packages/ggplot2/index.html).

### Determination of tumor immune biomarkers based on gene expression data

TIMER2.0 (19) was used to estimate tumor immune cell infiltration. TIMER, CIBERSORT (20), quanTIseq (21), xCell (22), McP-counter (23), and EPIC algorithms were used to estimate the gene expression profiles of colon cancers in high- and low-risk score groups. The immune infiltration score was represented as a heatmap with “ComplexHeatmap” package. GraphPad Prism 8.4 was used to comparatively estimate immune infiltration between high- and low-risk score groups.

### Genetic variation analysis

Mutation analysis of NRGs in colon cancers was performed with “maftools” (https://bioconductor.org/packages/release/bioc/html/maftools.html) software package between low- and high-risk score groups to screen different mutation genes. Mutation sites or prognosis-related mutation genes were also determined.

### Sensitivity analysis of chemotherapeutic drugs

The semi-inhibitory concentration (IC50) of antagonist indicates the inhibition degree of some physiological processes by a drug or inhibitor. IC50 value of a chemotherapy drug was predicted with “oncoPredict” (https://mirrors.sjtug.sjtu.edu.cn/cran/web/packages/oncoPredict/index.html) software package. A low IC50 value (toward 0) indicates the potent and effective role of the drug. Whereas, high IC50 value indicates that the drug is less effective. The expression matrix and drug processing information for the training set were obtained from the Genomics of Drug Sensitivity in Cancer database (https://www.cancerrxgene.org/). The expression matrix was GDSC2_Expr = readRDS (“GDSC2_Expr (RMA Normalized and Log Transformed).rds”). Drug processing information was GDSC2_Res = readRDS(“GDSC2_Res.rds”). The transcriptomic data of colon cancers in TCGA database were increased by 1 per value, followed by a logarithmic transformation with a base of 2. Differences in IC50 values of chemotherapy drugs between low- and high-risk score groups were analyzed. IC50 values with p < 0.05 and log FC>0.1 or <−0.1 were included in subsequent t-test of unpaired data to further compare IC50 of drugs between low- and high-risk score groups.

### Statistical analysis

Differentially mutation genes were identified with chi-squared test. The quasi-likelihood method was used to analyze DENRGs of RNA-seq data. Kaplan-Meier survival curves were analyzed with log-rank test. The t-test was used to compare mean values of unpaired data. All statistical significance was set as p < 0.05.

## Results

### General information of the study cohort

In total, 124 NRGs were obtained from KEGG database. The clinical data from 447 colon cancer patients were included in this study (**Figure S1**). Among them, 238 (53.2%) and 209 (46.8%) cases were aged < 70 years and >= 70 years, respectively (**Supplementary Table 1**). The transcriptomic data of 124 NRGs of 464 colon cancer samples and 41 control samples were obtained from TCGA database (**Supplementary Table 2**). Mutation data of 124 NRGs of 333 colon cancer patients were downloaded from TCGA database.

### Determination of DENRGs in colon cancers

**Figure 1:**
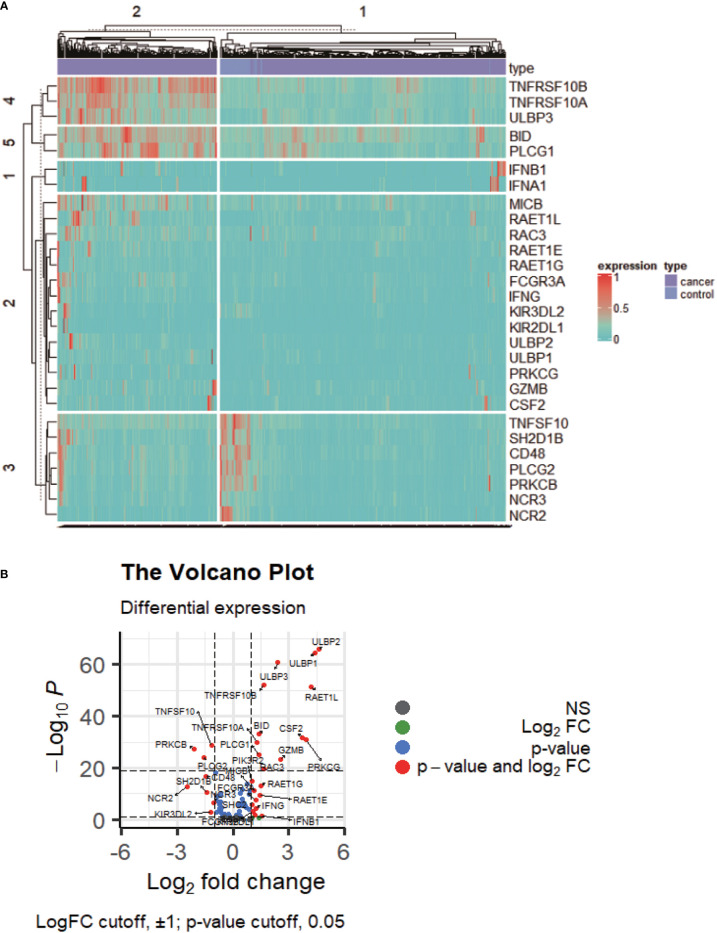
The expression profile of NKCP genes. **(A)**: Heat map of NKCP gene expressions in colon cancer normal control group vs cancer tissues. **(B)**: Volcanic map of NKCP gene expressions.

DENRGs were identified in colon cancers compared to controls, including 20 upregulated and 8 downregulated DENRGs (**Figures 1A, B**; p < 0.05, **Supplementary Table 3**). The 20 up-regulated DENRGs were ULBP2, ULBP1, RAET1L, PRKCG, CSF2, GZMB, ULBP3, RAC3, TNFRSF10B, RAET1G, IFNB1, BID, RAET1E, PLCG1, IFNA1, TNFRSF10A, FCGR3A, IFNG, MICB, and KIR2DL1. The eight down-regulated DENRGs were NCR2, PRKCB, PLCG2, CD48, SH2D1B, TNFSF10, KIR3DL2, and NCR3.

### DENRG ULBP2 as biomarkers of poor prognosis

**Figure 2:**
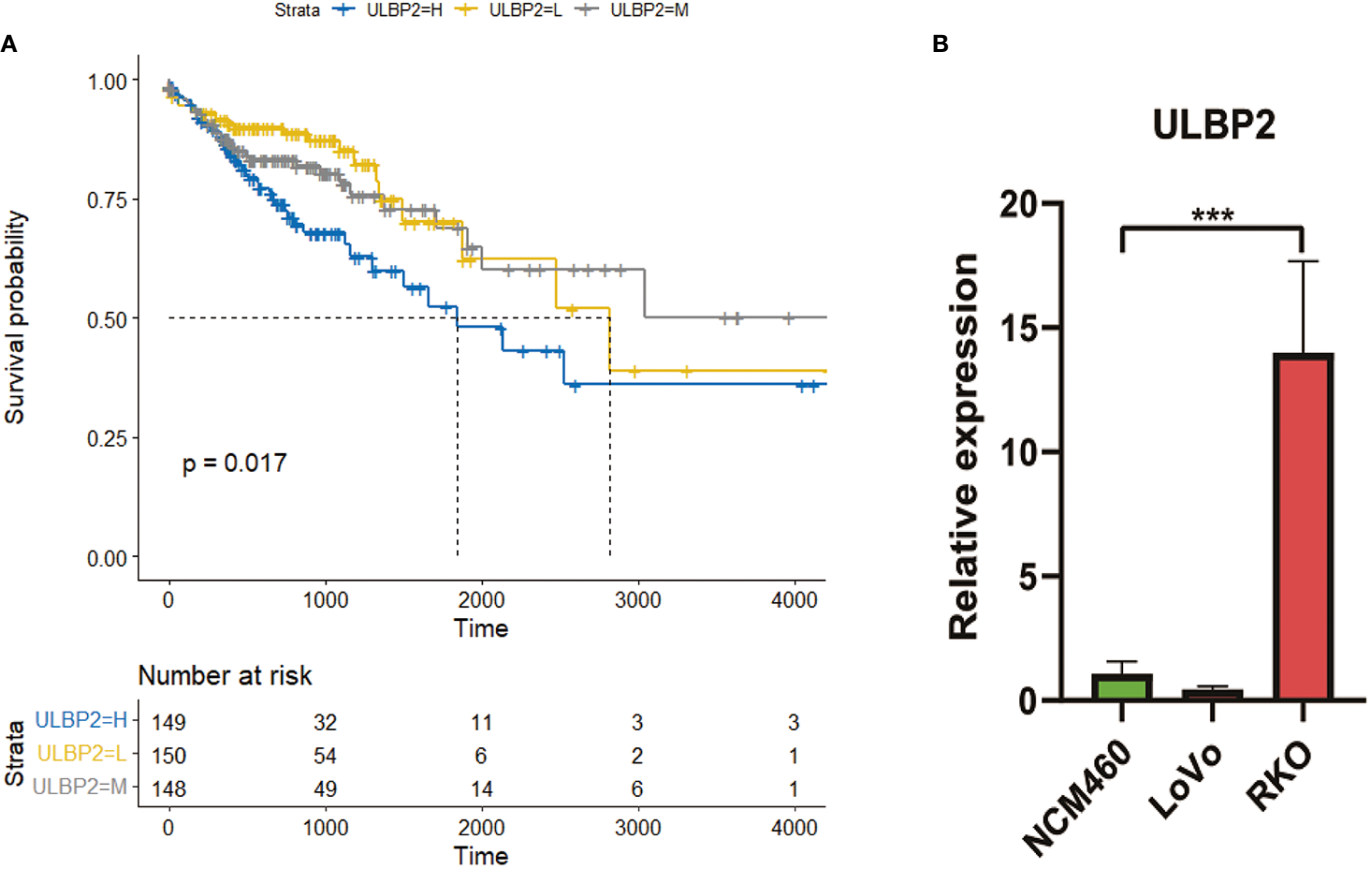
The expression and survival analyses of ULBP2. **(A)**: Survival analysis of ULBP2 among low-, medium-, and high-expression subgroups of ULBP2. **(B)**: Expression of ULBP2 in normal colon cell lines vs colon cancer cell lines quantified with qPCR.

Univariate analysis revealed that the survival of patients was longer in low-expressed group and medium-expressed group of ULBP2 compared to high-expressed group of ULBP2 (p < 0.05; **Figure 2A**; **Table S1**). No statistical difference was found in survival analysis of the other 27 DENRGs; for example, survival analysis of ULBP1 found no statistical difference between low-, medium-, and high-expression subgroups (p > 0.05, **Figure S2A**).The prognosis of patients aged < 70 years was better than that of patients aged ≥ 70 years (p < 0.05, **Figure S2B**). In addition, the prognosis of patients with stages I and II was better than that of patients with stages III and IV (p < 0.05, **Figure S2C**).

qPCR revealed that mRNA level of ULBP2 was higher in colon cancer cells RKO compared to control cells NCM460 (p < 0.05; **Figure 2B**); however, no statistical difference was found in colon cancer cells LoVo compared to control cells NCM460.

**Figure 3:**
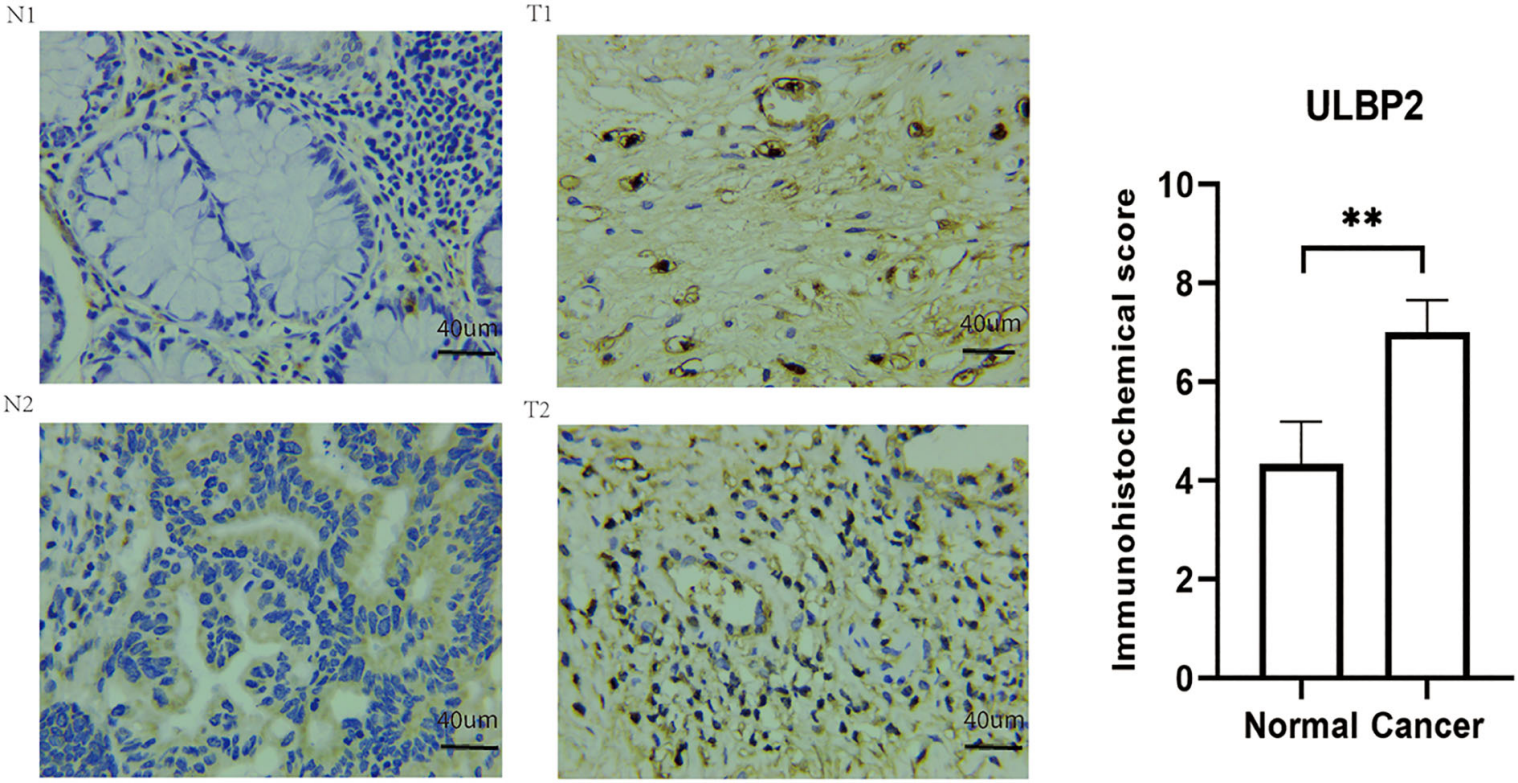
Immunohistochemical staining of ULBP2 in colon cancer tissues and adjacent healthy tissues. N: adjacent normal tissue, T: colon cancer tissue. ** P < 0.01.

The immunohistochemical analysis revealed that the protein level of ULBP2 was higher in colon cancer tissues compared to adjacent control tissues (p < 0.05, **Figure 3**). Similar results were also found in The Human Protein Atlas online the negative immunohistochemical staining of ULBP2 protein in two cases of normal colon tissues (**Figure S3**).

### Establishment and verification of the logistic regression model

Six statistically significant features were included in the established logistic regression model (**Supplementary Table 4**): S = 1.70 × stage – 9.32 × cg06543087 + 6.19 × cg25848557 + 1.29 × IFNA1 + 0.048 × age + 4.37 × cg21370856 − 8.93. The discriminated ability of each feature was shown (**Figure S4**). The clinical stage of colon cancers was the most important factor for survival. The accuracy of the logistic regression model (verified with 10-fold cross-validation) was 67.24% when it was established with only clinical stages to predict 3-year survival, and the area under the receiving operating characteristic curve (AUC) and the precision were 0.66 and 0.665, respectively. And the accuracy of the logistic regression model (verified with 10-fold cross-validation) was 59.48% when it was established with ULBP2 to predict 3-year survival, and the AUC and the precision were 0.657 and 0.502, respectively. The accuracy of the logistic regression model (verified with 10-fold cross-validation) was 70.69% when it was established with clinical stage and age to predict 3-year survival, and the AUC and the precision were 0.743 and 0.701, respectively. Moreover, the accuracy of the logistic regression model (verified with 10-fold cross-validation) was 80.17% when it was established with risk scores to predict 3-year survival, and the AUC and the precision were 0.855 and 0.801, respectively.

### Survival analysis of low- and high-risk score subgroups of colon cancers

**Figure 4:**
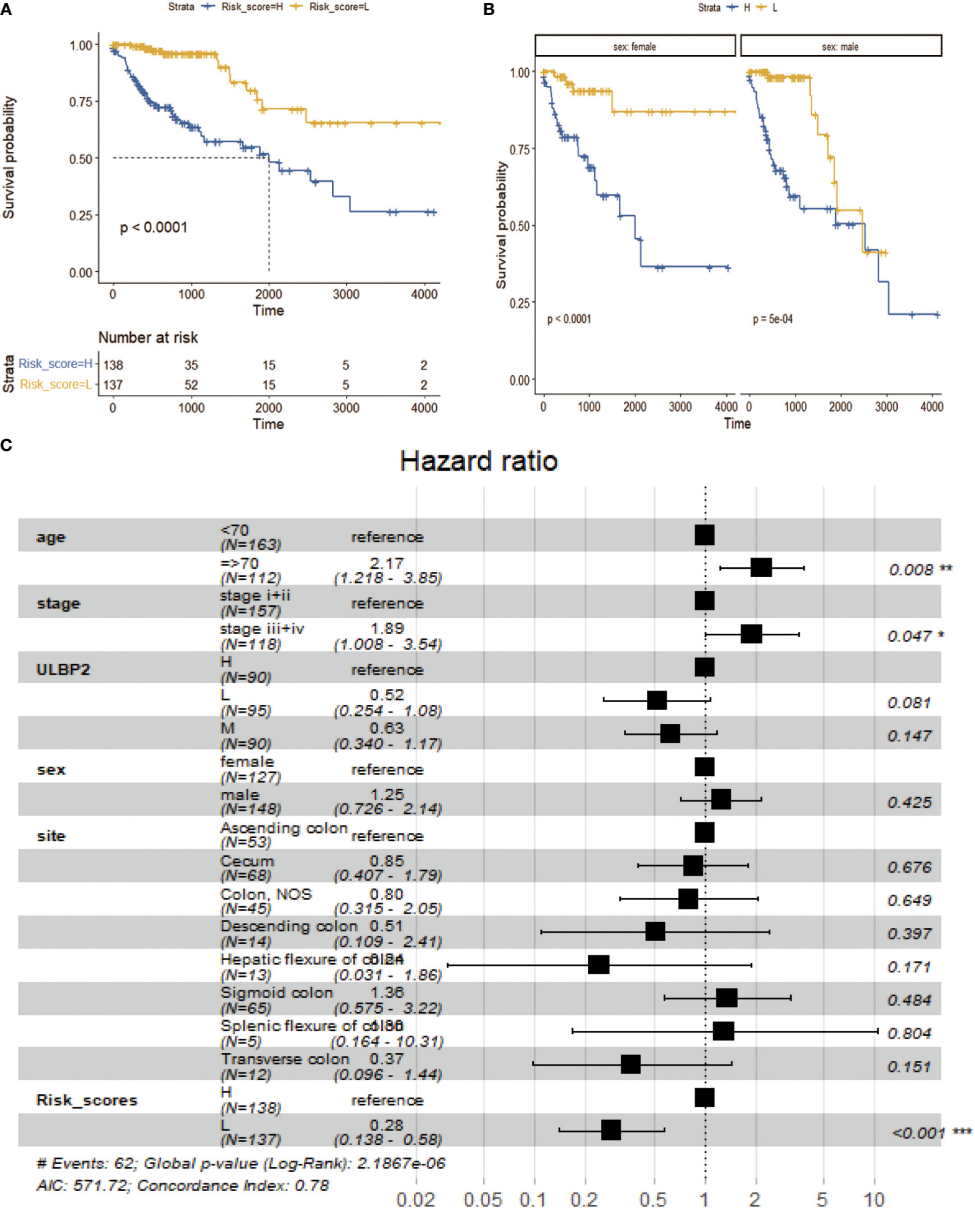
Survival analysis between low- and high-risk groups. **(A)**: Survival analysis of low-risk group vs high-risk group. **(B)**: Survival analysis of low-risk group vs high-risk group in different sex subgroups. **(C)**: Survival analysis of multivariate Cox risk model.

Kaplan-Meier survival analysis revealed that the prognosis of patients was significantly better in low-risk score group compared to high-risk core group (p < 0.0001; **Figure 4A**, **Supplementary Table 4**). When these patients were stratified into male and female subgroups, the prognosis of patients was still better in low-risk score subgroup than high-risk score subgroup in male and female, respectively (p < 0.001; **Figure 4B**). Multivariate Cox regression analysis revealed that age ≥ 70 years, late stage (stage iii+iv) and high-risk scores were risk factors of poor prognosis (**Figure 4C**, p < 0.05).

### Functional characteristics of DENRGs in colon cancers

**Figure 5:**
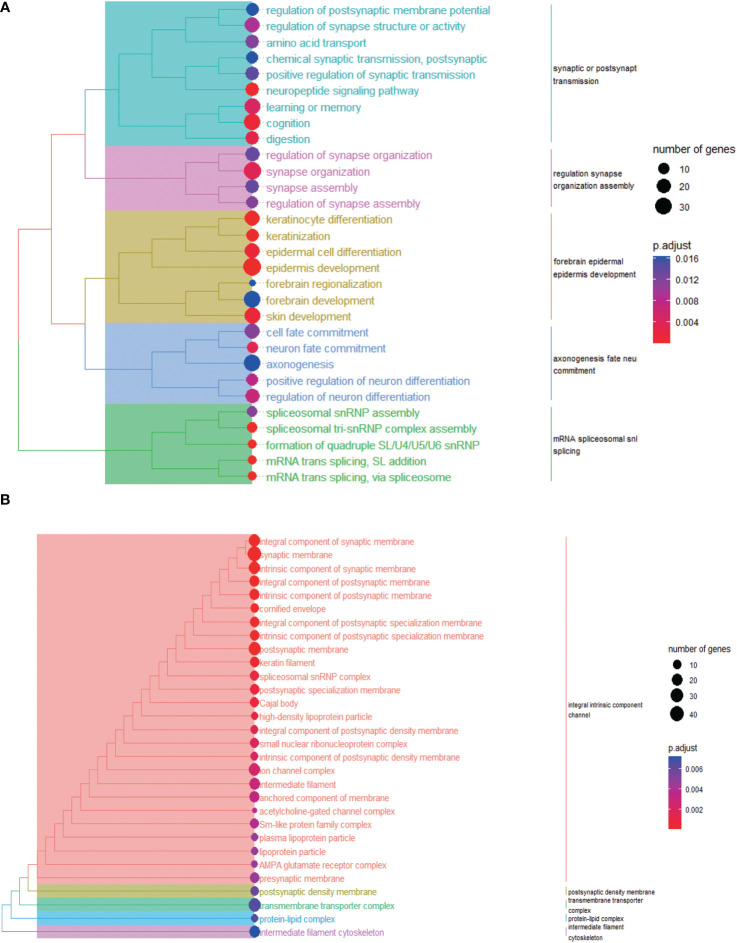
Biological process **(A)** and cellular component **(B)** enrichment analyses of differential genes between low-risk group and high-risk group.

GO enrichment analysis revealed that DENRGs (**Supplementary Table 5**) were enriched in two biological processes, including epidermis development and synapse organization (**Figure 5A**), two cellular components, including synaptic membrane and postsynaptic membrane (**Figure 5B**); and two molecular functions, including receptor-ligand activity and signaling receptor activity (**Figure S5A**). Also, KEGG pathway analysis revealed that DENRGs were mainly enriched in neuroactive ligand-receptor interactions and pancreatic secretions (**Figure S5B**).

### Tumor immune infiltration difference between high- and low-risk score groups of colon cancers

**Figure 6:**
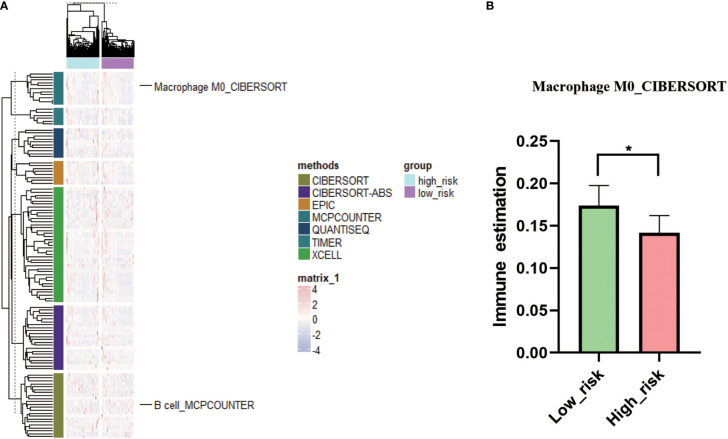
Immune status analysis in low- and high-risk groups. **(A)**: Complex heat map of immune estimation in low-risk group vs high-risk group. **(B)**: Comparison of immune estimates of macrophage M0 in low-risk group vs high-risk group. * P < 0.05.

Tumor immune analysis showed that immune infiltration score of M0 macrophages was significantly higher in low-risk score group compared to high-risk score group (**Figures 6A, B**; p < 0.05, **Supplementary Table 6**). Other common immune cell infiltration scores were not significantly different between high- and low-risk score groups.

### NRG mutation differences between high- and low-risk score groups of colon cancers

**Figure 7:**
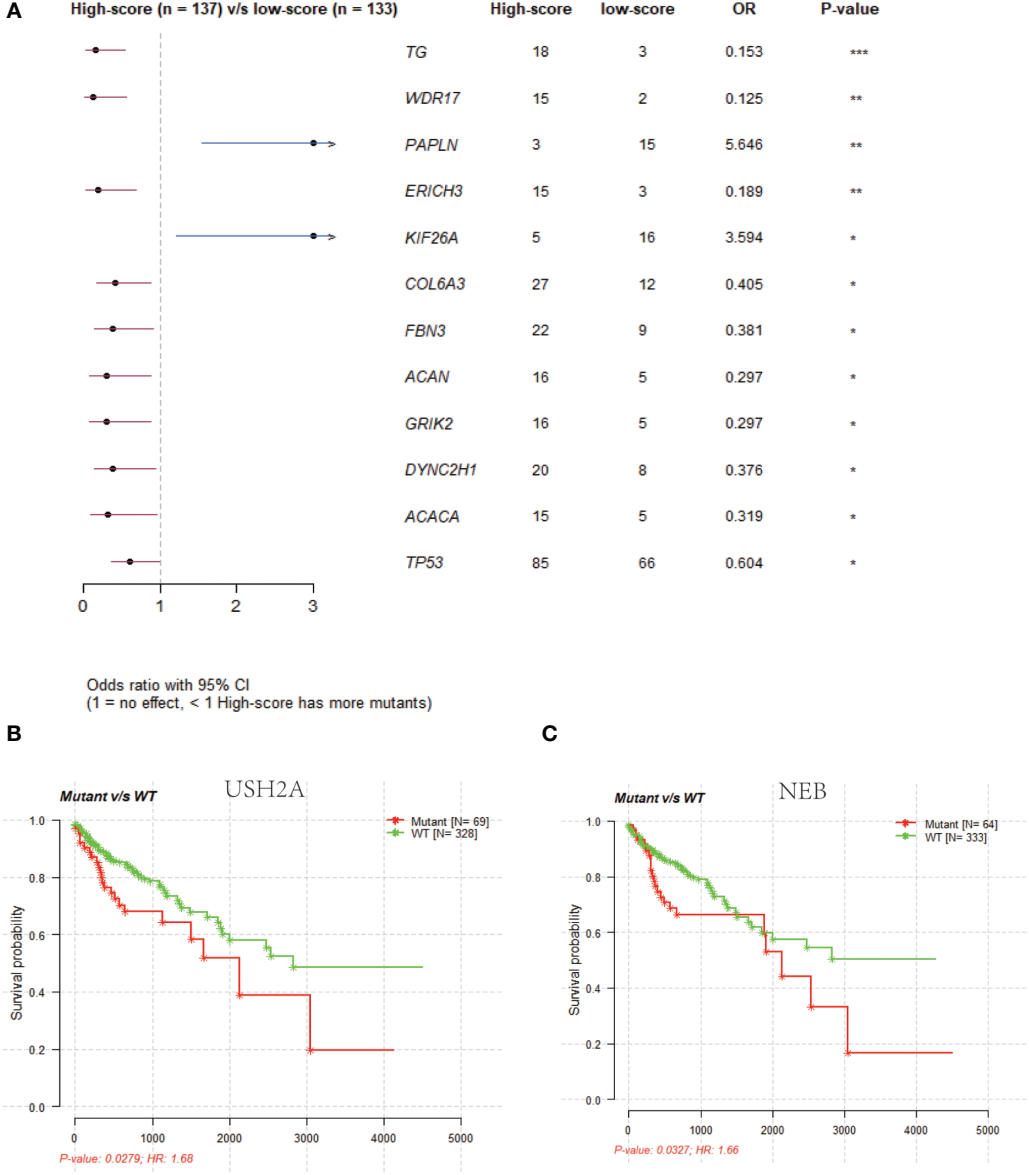
Gene mutation status analysis in low- and high-risk groups. **(A)**: Comparison of gene mutation rates in the low-risk (score) and high-risk (score) groups. **(B)**: Survival analysis of the USH2A wild-type and mutant groups. **(C)**: Survival analysis of the NEB wild-type and mutant groups.

The mutation incidence of *TG* [odds ratio (OR): 0.153; p < 0.05] and *WDR17* (OR: 0.125; p < 0.05) was higher in high-risk score group (**Supplementary Table 7**) compared to low-risk score group (**Supplementary Table 8**, **Figure 7A**). However, the mutation incidence of *PAPLN* (OR: 5.646; p < 0.05) and *KIF26A* (OR: 3.594; p < 0.05) was higher in low-risk score group than high-risk score group (**Figure 7A**). The survival time was longer in patients with wild-type *USH2A* (**Figure 7B**) and *NEB* (**Figure 7C**) compared to patients with mutant *USH2A* and *NEB* (p < 0.05). Among 12 NRGs with mutation ratio that was most significantly different between high- and low-risk score groups, 10 mutated NRGs were derived from high-risk score group (**Figure 7A**). The median tumor mutation burden was 2.2/MB (log10) in low-risk score group (**Figure S6**) and 2.34/MB (log10) in high-risk score group (p < 0.05).

### Chemotherapeutic drug sensitivity difference between low- and high-risk score groups of colon cancers

**Figure 8:**
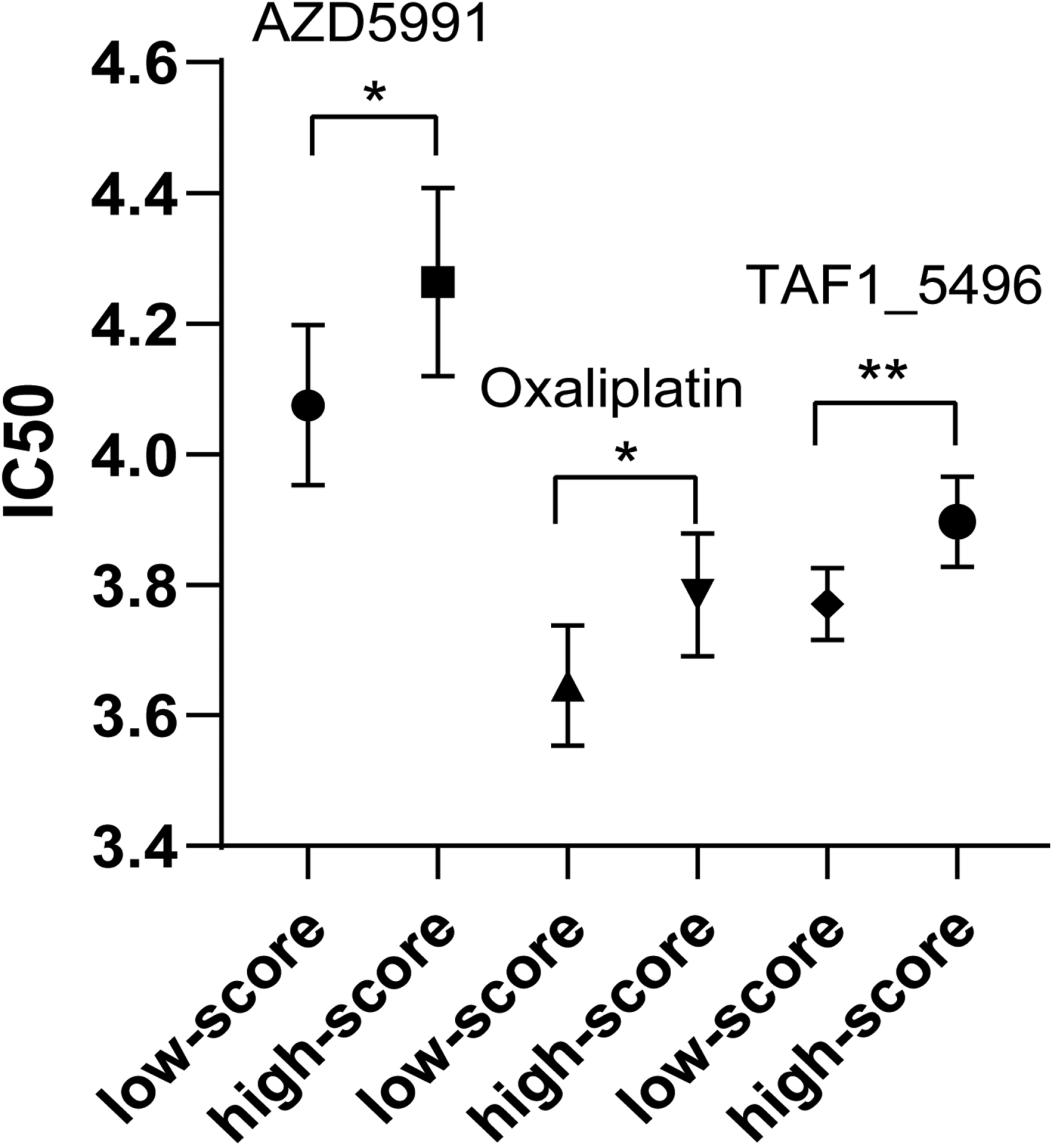
Comparison of IC50 of three chemotherapeutic drugs between low-risk group and high-risk group. * P < 0.05. ** P < 0.01.

Chemotherapeutic drug sensitivity analysis revealed that IC50 values of three drugs (AZD5991, oxaliplatin, and TAF1_5496) were higher in high-risk score group compared to low-risk score group (**Supplementary Table 9**, **Figure 8**; p < 0.05). No chemotherapeutic drugs exhibited higher IC50 values in low-risk score group relative to high-risk score group.

## Discussion

This study identified an NKCP-derived biomarker for colon cancer poor prognosis. The 3-year survival time can be predicted with logistic regression model that includes gene expression and methylation sites of NRGs and clinical information.

This study found that a NRG, ULBP2, was highly expressed at the mRNA level in colon cancer tissues and cell line. Immunohistochemical analysis also show ULBP2 was highly expressed in colon cancer tissues, which was consistent with previous immunohistochemical report in colon cancer tissues (24). Other studies found that ULBP2 was negatively regulated by miR-873 to inhibit proliferation, invasion, and migration of cervical cancer cells (25) and ULBP2 was bond by miR-6071 to inhibit glioma cell proliferation and invasion and promote cell apoptosis through PI3K/Akt/mTOR pathway (26). The immunoligand ULBP2:HER2-scFv can trigger NK cell cytotoxicity against tumors and boost antibody-dependent, cell-mediated cytotoxicity (27). Thereby, ULBP2 in many malignant tumors, including colon cancer, is a potential clinical diagnostic and therapeutic biomarker.

Currently, it is not accurate for the clinical survival prediction of colon cancer patients. The analyses of NKCP and clinical information revealed that tumor clinical stage was the most significant factor to predict 3-year survival of colon cancer patients. However, this study found that the prediction accuracy only based on tumor clinical stage was only 69.83% for 3-year survival prediction. When ULBP2 was used alone to distinguish 3-year survival time, the accuracy of logistic regression model was only 59.48%.One study used immune score to predict 3-year prognosis and survival of colon cancer patients, which achieved the AUC values 0.754 in training group and 0.691 in validation group (15). Thereby, a more comprehensive, effective prediction model is needed to predict the 3-year survival of colon cancer patients. This present study established a logistic regression model with six features, including two clinical features (tumor clinical stage and patient age), one NRG, and three methylation site data of NRGs. Of the six features, tumor clinical stage was the crucial factor to predict the prognosis and survival of colon cancer patients. Also, NRG methylation sites have potential contribution to the prognosis and survival of colon cancer patients. The logistic regression model-based risk scores divided colon cancer patients into high- and low-risk score groups. The prediction accuracy of 3-year survival of colon cancer patients based on this prognostic model was 80.17% (10-fold cross-validation), which was significantly higher than clinical stage-based prognostic model and other models (13–16).

Univariate and multivariate Cox analyses revealed that risk score derived from this six-feature logistic regression model was a good indicator of prognosis. It clearly demonstrates that this risk score after dimensionality reduction is a biomarker of poor prognosis, and can efficiently predict 3-year survival time.

The proportion of macrophages was significantly different between colon cancers and controls. A high proportion of M0 macrophages is associated with low-risk score group of colon cancers (28). The number of M0 macrophage was higher in high-risk score group relative to low-risk score group of colon cancer patients (29), whereas for liver cancer patients, the level of M0 macrophage was significantly downregulated in high-risk score group (30). However, this study found that M0 macrophage infiltration was higher in low-risk score group compared to high-risk score group. These inconsistent results might be resulted from different experimental conditions in different studies.

Gene mutational analysis of NRGs revealed that the median mutation burden was higher in high-risk score group compared to low-risk score group in colon cancer patients. In addition, NRGs with the most significant mutation rate were mainly distributed in high-risk score group, which suggests that NRG mutations are likely associated with poor prognosis of colon cancer. Study found that the high mutation burden predicted the poor prognosis of colorectal cancers (31). Also, the increased tumor mutation burden occurred in high-risk group of lung cancers (32), and was associated with poor prognosis of colon cancers (33).

Furthermore, drug sensitivity analysis evidenced the positive effect of the risk score based on six-feature logistic regression model of colon cancers. IC50 value is an effective indicator for drug sensitivity, and lower IC50 means better drug sensitivity. IC50 values of three chemotherapeutic drugs were higher in high-risk score group compared to low-risk score group. A study also found that cancer patients in high-risk group were less sensitive to multiple chemotherapy agents (29). Interestingly, for oxaliplatin as the first line of treatment for colon cancer (34), its IC50 value was higher in high-risk score group compared to low-risk score group in colon cancers. The cancer patients who have good sensitivity to chemotherapeutic drugs can be often effectively controlled, which results in an improved prognosis. It is reported that the inhibitory concentration of oxaliplatin was ~4.2 μM in colon cancer cells (35), which was similar to its IC50 value identified in this study.

### Limitations and prospects

The six features that were included in this logistic regression model were mainly derived from clinical information and NKCP. The prediction accuracy of six-feature logistic regression model for 3-year survival prediction might be further improved if a large range of genes and methylation sites are used for dimensionality reduction analysis. In clinical practice, if NRG sequencing data are not available from patients, this logistic regression model developed in this study can be used to roughly predict 3-year prognostic survival based on patient age and tumor stage, with a prediction accuracy of ~70%. If clinical doctors can obtain NRG sequencing data and methylation sites, the prediction accuracy of this logical regression model for 3-year survival prediction is able to be over 80%.

## Conclusions

NKCP is significantly associated with colon cancer, and ULBP2 derived from NKCP is a poor prognosis biomarker of colon cancer. The established six-feature logistic regression prognostic model involved mRNA expression data and methylation site of NRGs in combination with patient age and tumor stage, which can effectively calculate the risk score for each patient. This risk score was a poor prognosis biomarker of colon cancer patients, and can effectively predict 3-year survival rate of colon cancers. Compared to high-risk score group of colon cancers, the colon patients in low-risk score group had better prognosis, including longer survival time, higher M0 macrophages infiltration score, lower tumor mutation burden, and lower IC50 value of chemotherapeutic drugs.

## Data availability statement

The datasets presented in this study can be found in online repositories. The names of the repository/repositories and accession number(s) can be found in the article/**Supplementary Material.**

## Ethics statement

The studies involving human participants were reviewed and approved by the ethics committee of Shandong First Medical University, Ethics No. 202201170002. The patients/participants provided their written informed consent to participate in this study.

## Author contributions

ZY analyzed data, prepared figures and tables, and wrote manuscript. HZ and ZY performed qPCR and immunohistochemical experiments. JL collected colon cancer tissues. XZ and SY conceived the concept, designed and critically revised the manuscript, and were responsible for its corresponding works. All authors contributed to the article and approved the submitted version.

## Supplementary material

Supplementary Figure 1 Mulberry plot of clinical information of patients with colon cancer.

Supplementary Figure 2 Survival analysis of patients. **(A)**: Survival analysis of ULBP1 in low-, medium-, and high-expression subgroups of ULBP1. **(B)**: Survival analysis of patients with colon cancer at different ages. **(C)**: Survival analysis of colon cancer patients with different clinical stages.

Supplementary Figure 3 Immunohistochemical staining of ULBP2 in two normal colon tissues (source: Human Protein Atlas; www.proteinatlas.org).

Supplementary Figure 4 The importance of six eigenvalues in distinguishing 3-year survival.

Supplementary Figure 5 Enrichment analysis of molecular function **(A)** and KEGG pathways **(B)** of DENRGs

Supplementary Figure 6 Tumor mutation burden in the low-risk group vs high-risk group.
