# Supplementary figures and images for: Significance of logistic regression scoring model based on natural killer cell-mediated cytotoxic pathway in the diagnosis of colon cancer
Source: Front Immunol. 2023 Jan 20;14:1117908. doi: 10.3389/fimmu.2023.1117908 (PMC9895796; doi:10.3389/fimmu.2023.1117908)

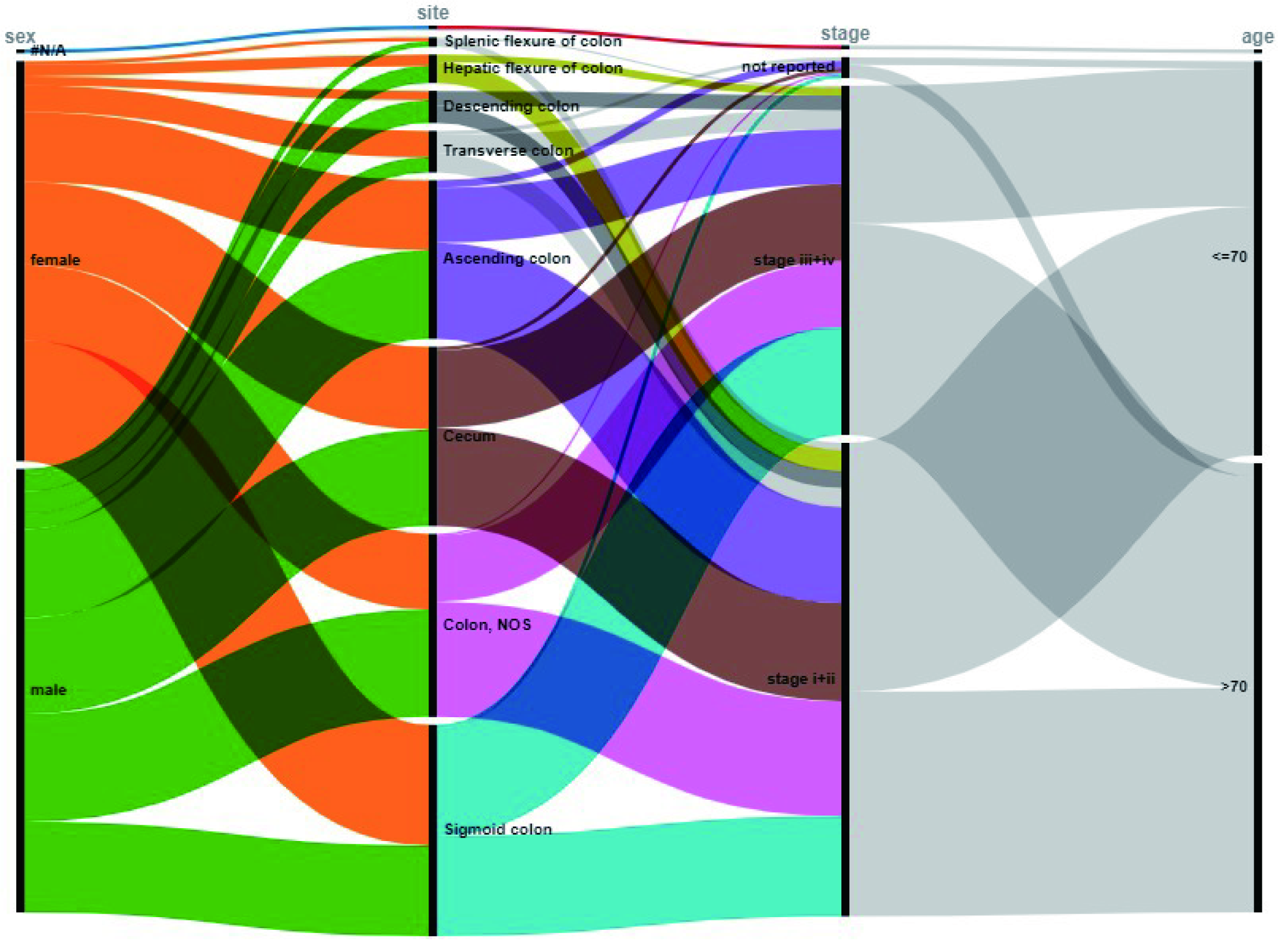

Supplement: Supplementary Figure 1 — Mulberry plot of clinical information of patients with colon cancer. [file Image_1.tif]

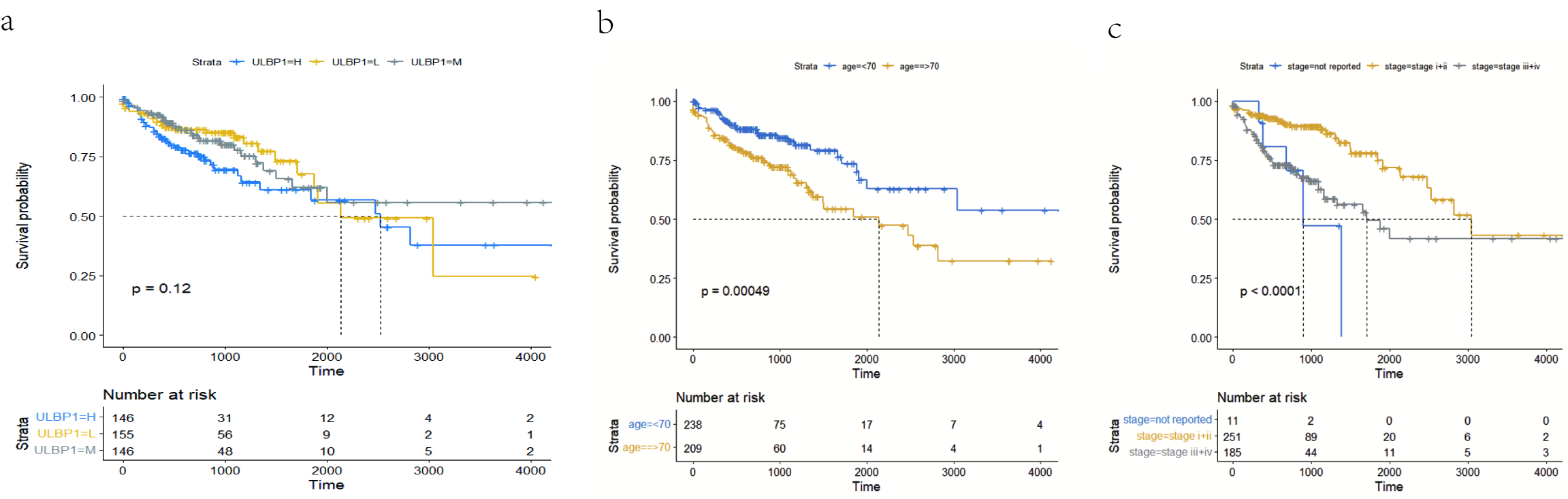

Supplement: Supplementary Figure 2 — Survival analysis of patients. (A): Survival analysis of ULBP1 in low-, medium-, and high-expression subgroups of ULBP1. (B): Survival analysis of patients with colon cancer at different ages. (C): Survival analysis of colon cancer patients with different clinical stages. [file Image_2.tif]

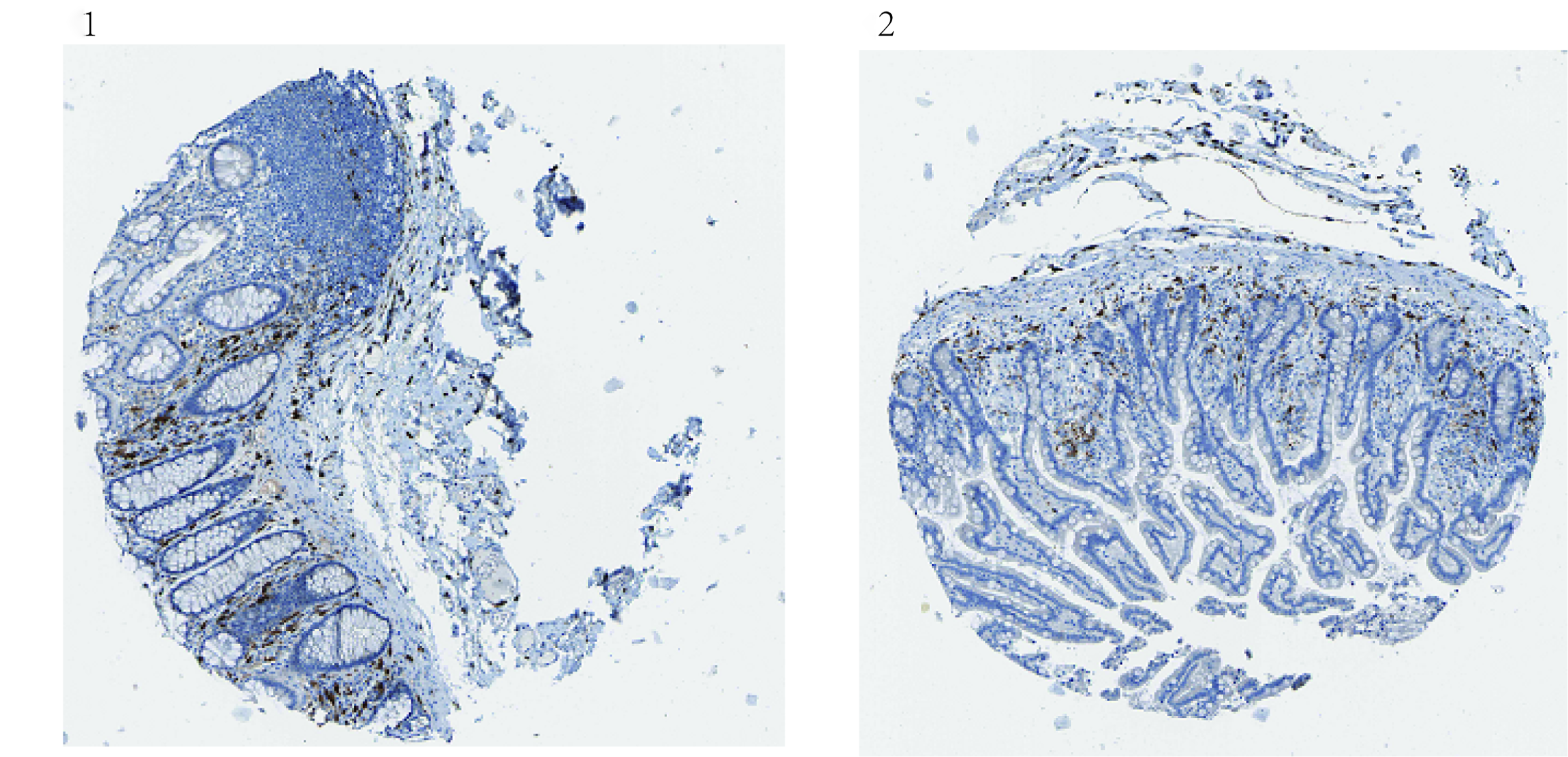

Supplement: Supplementary Figure 3 — Immunohistochemical staining of ULBP2 in two normal colon tissues (source: Human Protein Atlas; www.proteinatlas.org). [file Image_3.tif]

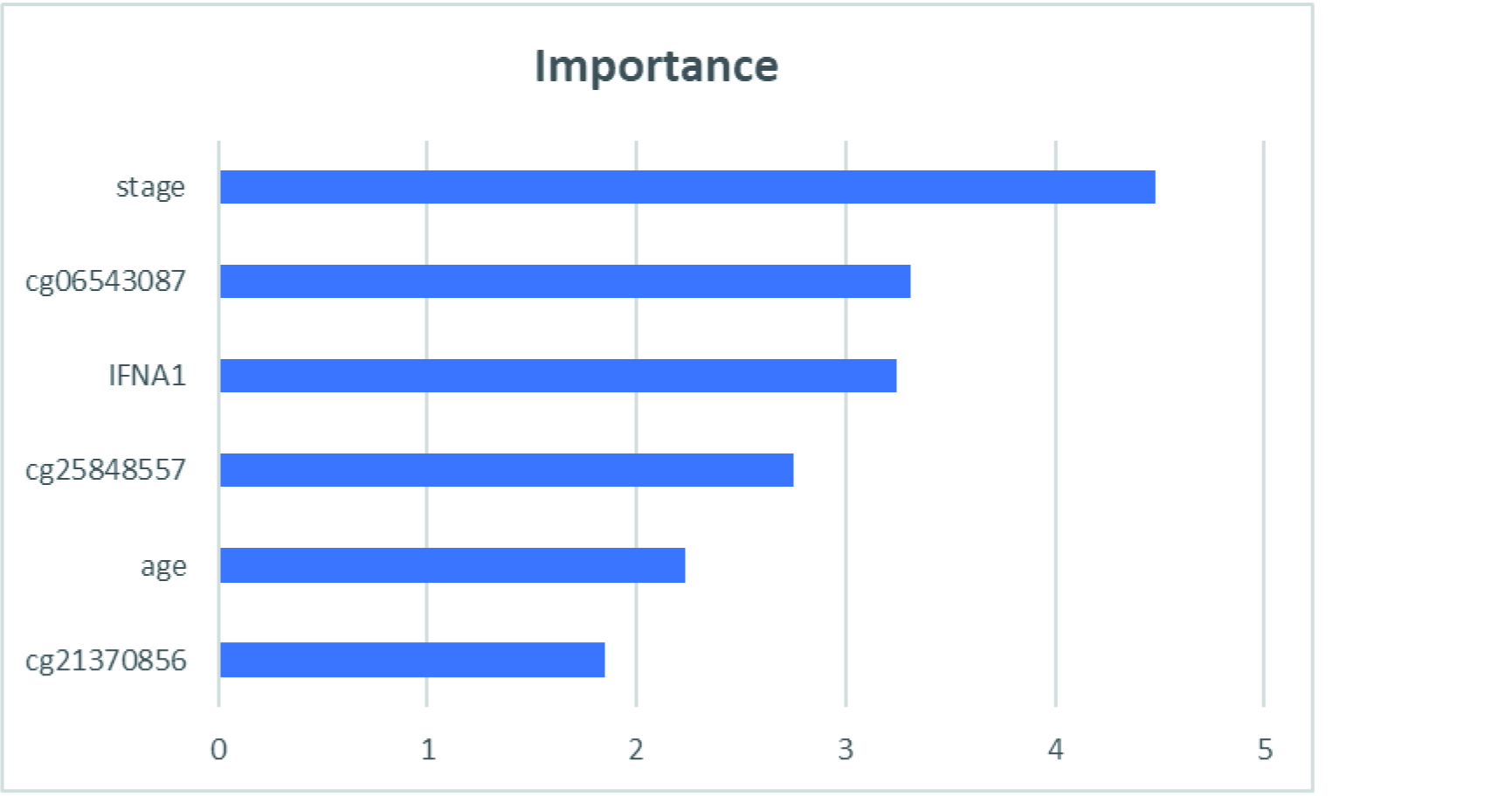

Supplement: Supplementary Figure 4 — The importance of six eigenvalues in distinguishing 3-year survival. [file Image_4.tif]

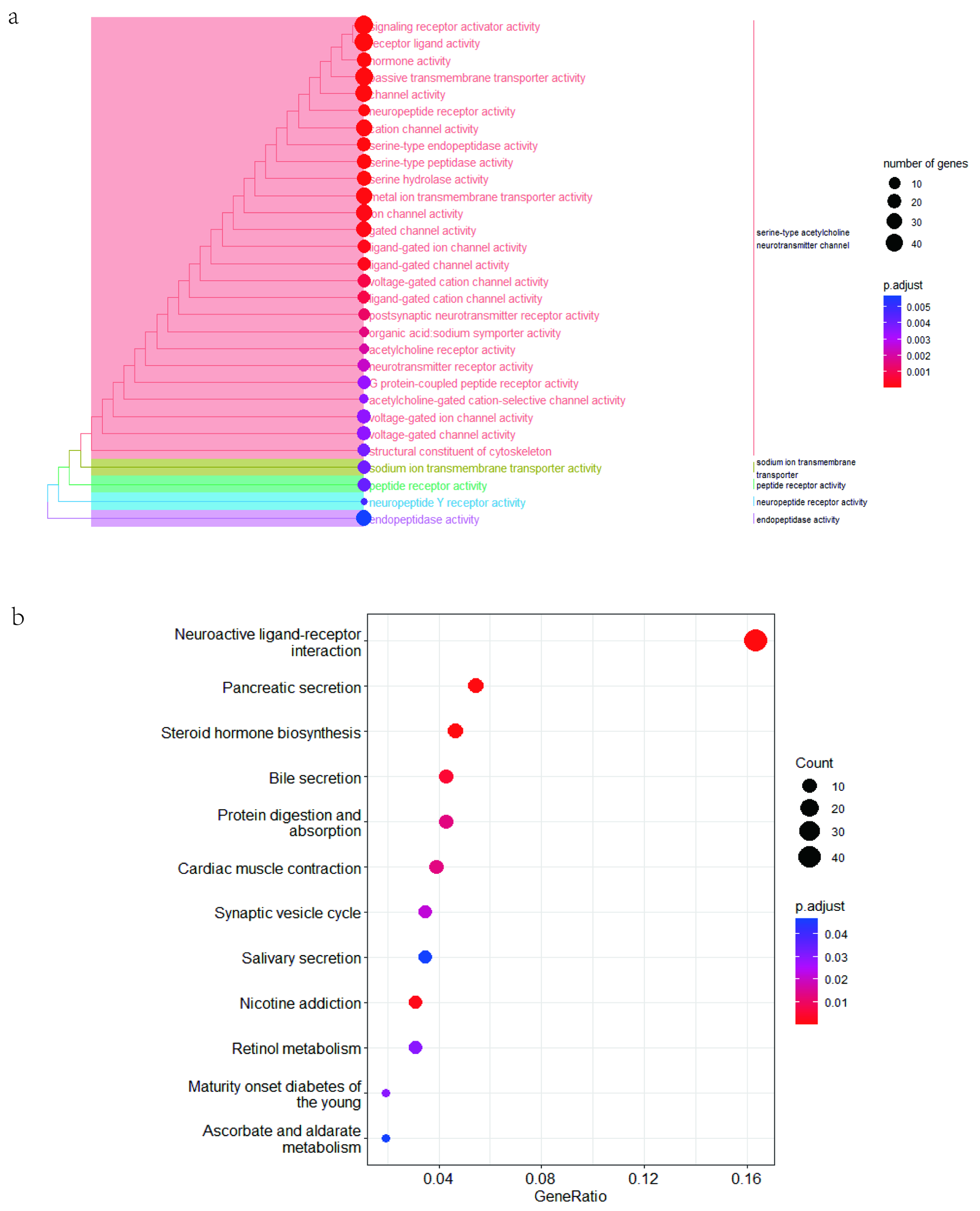

Supplement: Supplementary Figure 5 — Enrichment analysis of molecular function (A) and KEGG pathways (B) of DENRGs [file Image_5.tif]

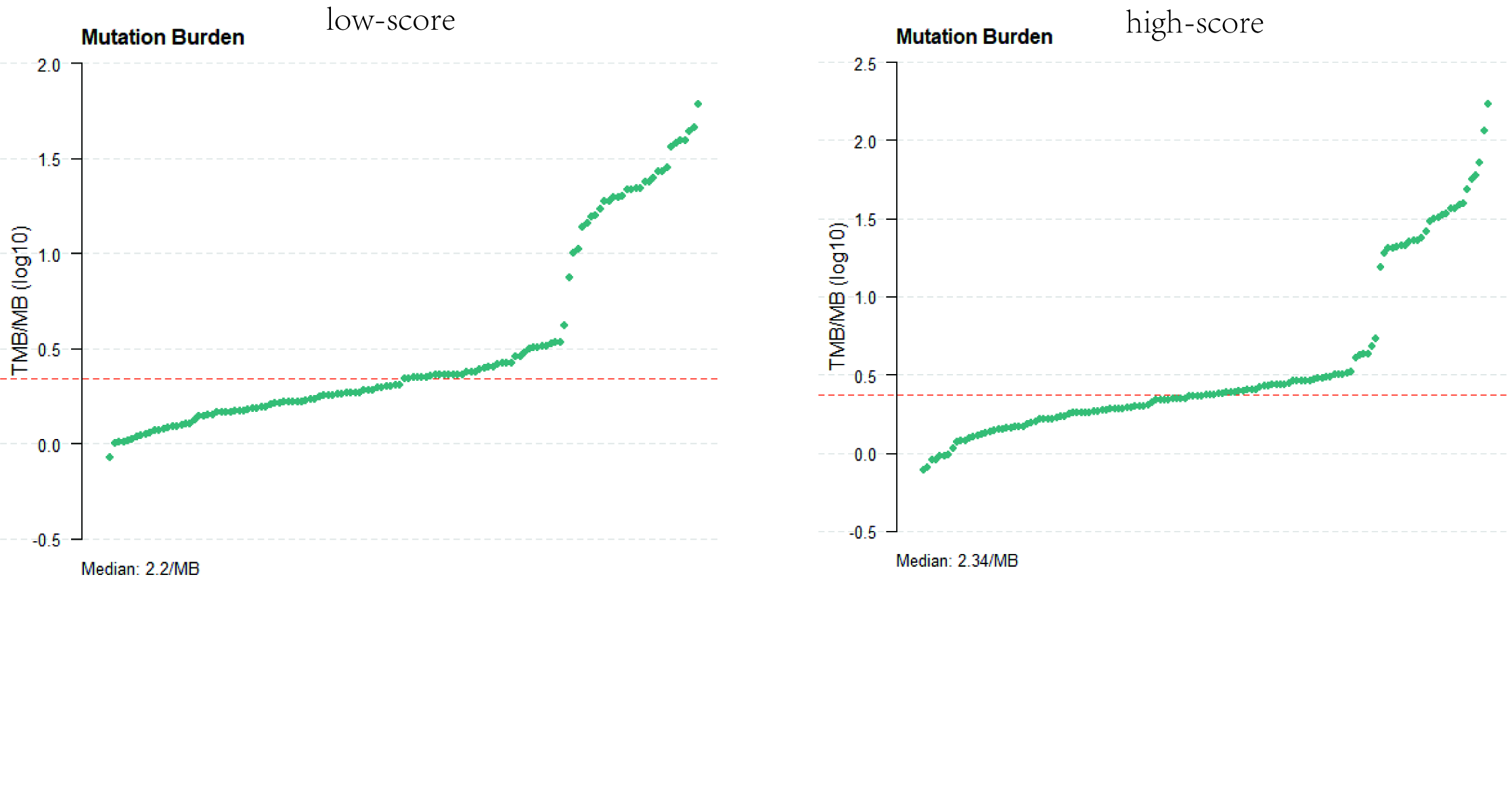

Supplement: Supplementary Figure 6 — Tumor mutation burden in the low-risk group vs high-risk group. [file Image_6.tif]
